# A shared basis for nutrient limitation response in cyanobacteria

**DOI:** 10.1016/j.jbc.2025.110765

**Published:** 2025-09-24

**Authors:** Hagit Zer, Stav Chen, David Rasin, Miguel Hernandez-Prieto, Nir Keren

**Affiliations:** 1 Institute of Life Sciences, The Hebrew University of Jerusalem, Jerusaelm, Israel; 2 Sydney Analytical, The University of Sydney, Sydney, Australia

**Keywords:** cyanobacteria, photosynthesis, chlorophyll, nutrient limitation, nitrogen, phosphate, sulfur

## Abstract

Cyanobacteria possess diverse regulatory mechanisms to adapt to nutrient limitation, yet the extent to which these responses are shared across different nutrient stresses remains unclear. Understanding these commonalities can reveal fundamental principles of cellular resource allocation and survival strategies. In this work, we investigated the transcriptional responses of *Synechocystis sp.* PCC 6803 to nitrogen, sulfur, or phosphate limitation and found a core set of genes consistently regulated across all three conditions. This shared response includes repression of genes related to photosynthesis and respiratory electron transport, as well as genes encoding components of the Calvin–Benson cycle, ribosome function, and cellular metabolism. Amongst the highest affected pathways is chlorophyll biosynthesis. A subset of regulatory genes, mostly kinases, are upregulated under all three limitation conditions. These results were further validated by a study of the composition and the function of the photosynthetic machinery. Chlorophyll accumulation was arrested immediately upon transition to limiting media, photosynthetic activities were reduced, and protein complexes were degraded. Our findings reveal a conserved program in cyanobacteria that modulates cellular metabolism and photosynthesis in response to diverse nutrient limitations. Based on these findings, we suggest that chlorophyll biosynthesis is a key regulated pathway driving structural and physiological responses in photosynthesis.

The bioavailability of mineral nutrients is a major factor shaping the distribution, composition, and function of photosynthetic organisms in both natural and agricultural settings. The macronutrients nitrogen (N), phosphorus (P), and sulfur (S) are required for building proteins, nucleic acids, and membranes, whereas micronutrients, such as iron (Fe), manganese (Mn), and copper (Cu), play specific roles as prosthetic groups and cofactors in the cellular energy metabolism (1, 2, 3). For planktonic algae and cyanobacteria, the bioavailability of nutrients can determine the rate, extent, and duration of a bloom cycle. Nutrient pollution and eutrophication can change the species composition and lead to detrimental results such as toxic cyanobacterial blooms (4).

The study of nutrient limitations in cyanobacteria uncovered a number of specific photosynthetic acclimation responses. A complex regulatory system has been developed during evolution, in which regulatory proteins, noncoding RNAs (ncRNAs), redox mediators, and small organic molecules serve as effectors to drive acclimation processes. For example, in nondiazotrophic cyanobacteria, N limitation promotes the degradation of the phycobilisome (PBS) antenna. This is preceded by the induction and accumulation of NblA proteins (5). The function of PBS degradation generates an amino acid pool for critical cellular functions and reduces photosystem (PS) excitation pressure in the absence of anabolic activity. S limitation similarly results in degradation of the PBS (6). The rate and extent of PBS degradation vary between different cyanobacterial species and limitation conditions (7).

Fe limitation also exerts a specific photosynthetic response. The Fe-induced *isiAB* operon codes for a membrane-embedded chlorophyll-binding protein, which assembles around photosystem I (PSI) complexes (IsiA) and a flavin-containing electron carrier flavodoxin (IsiB) that replaces the Fe–S–containing ferredoxins in the electron transport chain (8, 9). While the role of IsiB in reducing the Fe requirement is clear, the function of IsiA is still under debate. It was suggested that it is required for increasing the light-harvesting cross section of a diminishing PSI pool under Fe limitation and/or as a temporary reservoir for the chlorophylls released when PSI is degraded to repurpose its Fe centers (8, 10, 11, 12). Under Cu limitation, likewise, an Fe-containing electron carrier (cytochrome *c*) is induced to replace the Cu-containing plastocyanin in the photosynthetic electron transfer pathway (13). Mn limitation has also been tested (14). Mn is an essential part of the water-splitting complex within photosystem II (PSII), but its depletion had a major effect on PSI. PSI supercomplexes monomerized, and their content was reduced under Mn limitation (14).

Limitations of P bioavailability result in growth arrest and draw significant cellular changes. Its effect on the PBS is smaller than that of N or S limitation (7). However, P limitation increases the accumulation of cyanophycin, a multi-l-arginyl-poly-l-aspartate polymer, which can serve as an amino acid source (15). It is also well established that P limitation reduces the mRNA cellular content and the DNA copy number (16).

While in previous studies, we and other researchers often focused on the responses of cyanobacteria to specific nutrient limitation and the regulatory networks controlling these responses and their integration into cellular homeostasis, here, we set out to identify the common responses that occur once nutrient limitation starts affecting the culture survival. We do that by comparing gene expression profiles and photosynthetic performance in response to N, P, or S limitation in one well-studied cyanobacterial species, *Synechocystis sp*. PCC 6803.

## Results

**Figure 1:**
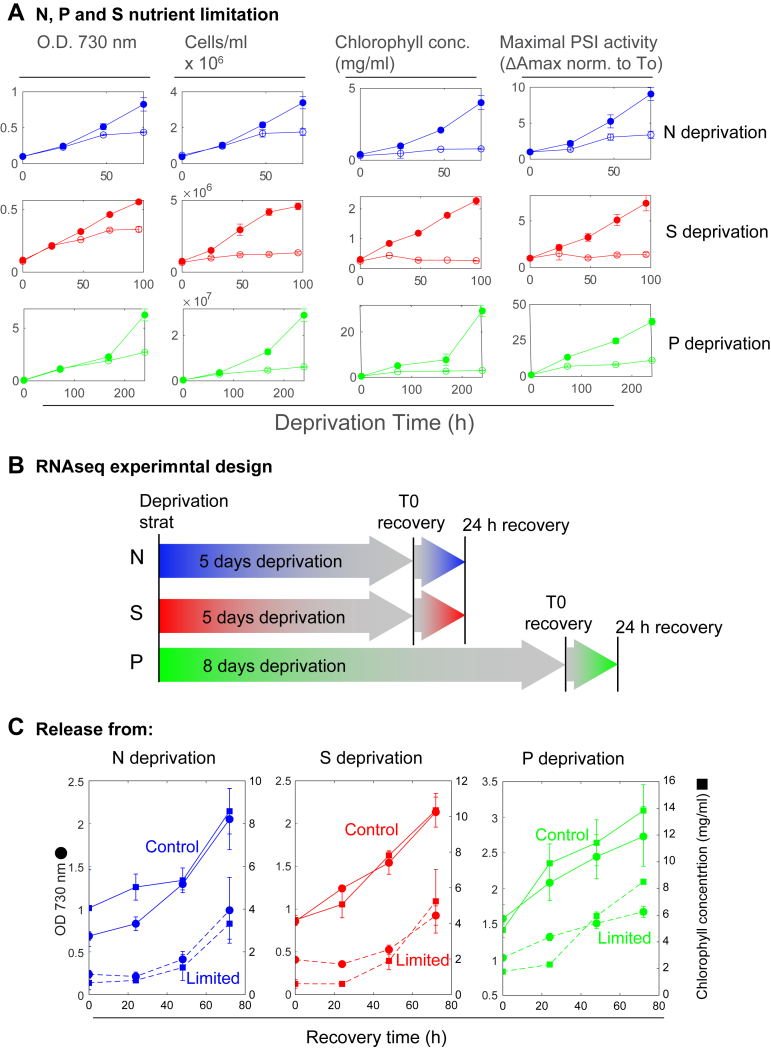
**Nutrient limitation conditions and recovery rates.***A, nutrient deprivation.* Cultures were transferred either into full control media (*full symbols*) or into media from which nitrogen (N), phosphorus (P), or sulfur (S) was completely removed (*empty symbols*). Error bars represent standard deviation (n = 3, biological replicates). The progression of each of the limitations and their corresponding control cultures was tracked to the point where complete growth arrest could be indicated. Cell growth was monitored as scattering at 730 nm and by cell counting. Chlorophyll concentrations were determined following methanol extraction. The maximal PSI activity was determined as ΔAmax as described (13). It was normalized to the value at T0 facilitate the comparison between the experiments. *B, design of the RNA-Seq experiment*. *Arrows* represent the time course of the nutrient deprivation period, T0 and T24 of the recovery period. The color scheme (*blue*—N, *green*—P, and *red*—S) is kept through the article. *C,* r*ecovery from nutrient deprivation*. Pairs of limitation (*dashed lines*) and control (*solid lines*) cultures are presented for each limitation scenario. Absorbance at 730 nm values are presented on the *left-hand axis* (*circles*). Chlorophyll concentrations (*squares*) are presented on the *right-hand axis*. Standard deviation values are based on n = 6 for N and S, n = 3 for P (biological replicates). PSI, photosystem I.

The first challenge in designing an RNA-Seq experiment was to establish a framework in which the three nutrient deprivation conditions could be compared. N, P, and S deprivations were induced by transferring *Synechocystis* 6803 cultures to media from which these elements were removed completely. As can be seen in Figure 1*A*, the measurable effects of N and S limitation progressed faster than the effect of P limitation (Fig. 1*A*). Growth of N- and S-starved cultures was arrested within 2 to 3 days, whereas P limitation required a much longer time to produce a significant difference in growth and chlorophyll concentration (Fig. 1*A*). Measuring the effect on RNA levels, as the limitation progresses, will require different time scales, which would complicate the analysis. We took a different approach. To equalize the conditions for a comprehensive analysis of shared response pathways, we designed an RNA-Seq experiment in which we compared limited cultures to each other at the end of the limitation period and 24 h after the addition of the limiting nutrient (Fig. 1*B*). RNA-Seq analysis was initiated using samples collected at the end of the nutrient limitation period (time 0), which corresponded to 5 days of -N and -S deprivation, or 8 days of -P deprivation (Fig. 1*B*). The recovery time point was taken 24 h after reintroducing the previously limiting nutrient. For all three limitation conditions, 24 h represents a time frame where the cells start dividing (Fig. 1*C*). Using this approach, we could compare transcript abundance of growth-arrested starved cells as compared with 24 h of recovery, within the same time frame for N, P, and S.

### Highly expressed genes under nutrient limitation conditions

A ranked list of the most highly expressed genes under nutrient deprivation (Fig. 1*B*: time 0) is available in Table S1. Regardless of whether these genes increased or decreased in expression following nutrient repletion, their high expression levels during limitation suggest a fundamental role in maintaining cellular viability under stress. The most highly expressed gene across all limitation conditions was the ncRNA *ssrA* (also known as tmRNA or 10Sa RNA (17)), with expression levels approximately sevenfold higher than the next most expressed gene. Together with the SmpB protein, *ssrA* is essential for the recycling of stalled ribosomes—an expected bottleneck under nutrient stress conditions such as N and S deprivation (which limit amino acid availability) or P starvation (which impairs ATP-dependent processes such as tRNA charging and ribosomal translocation).

Other highly expressed genes included components of the photosynthetic machinery. These encompassed PSII core subunits *psbA2* and *psbA3* (D1 protein), *psbD* and psbD2 (D2 protein), and *psbC* (CP43 internal antenna protein). Several PSI subunit genes were also among the top expressed, including *psaA*, *psaB*, *psaC*, and *psaF*, along with electron transport chain components such as *petE* (plastocyanin), *petD* (cytochrome b_6_f subunit 4), and *petB* (cytochrome *b*_6_). These genes are known to be constitutively expressed at high levels and often serve as internal controls in quantitative RT–PCR and Northern blot assays. For instance, *rnpB*, which encodes the RNA subunit of RNase P responsible for the processing of tRNAs, was the second most abundant transcript under all conditions.

### Differentially expressed gene categories

Differential expression was calculated as the log_2_ ratio of transcript abundance at 24 h postrecovery relative to time 0 (*i.e.*, log_2_[time 24 h/time 0 h]). Accordingly, positive values indicate increased expression during recovery, whereas negative values reflect reduced expression compared with the prerecovery state. These were classified into functional categories (Fig. S1). To differentiate between constitutively expressed genes and those transcriptionally regulated in response to nutrient stress, we focused on functional categories that exhibited consistent and significant expression change. This was defined as (a) an average fold change greater than twofold for the category (b) in all three nutrient limitation conditions. These categories are summarized in Table 1. For each category, an overall response score was calculated by summing the pairwise products of average fold changes across conditions, providing a composite metric of the consistency and magnitude of transcriptional regulation.

**Table 1:** Functional enrichment analysis of differentially expressed gene categories

Gene category	-N	-P	-S	Sum of products
PBS	3.94	1.62	4.39	30.79
PSI	3.46	1.42	2.79	18.53
CO_2_ fixation	2.9	1.74	2.88	18.41
Ribosomal proteins: synthesis and modification	1.87	2.5	1.91	13.02
PSII	2.24	1.77	2.09	12.35
Fatty acid, phospholipid, and sterol metabolism	1.65	1.6	1.59	7.81
Regulatory functions	−1.29	−1.49	−1.09	4.95

The table presents significantly affected functional categories among the three nutrient starvation conditions (-N, -P, and -S). The values represent the average log fold change of gene expression of the genes within that category for each starvation condition. The data were filtered using a *p*-adjusted value threshold of <0.05 per gene and a *p* value threshold of <0.05 per category. Only categories with log fold change values greater than 1 or less than −1 were included (equivalent to a fold change greater than 2). Functional enrichment was assessed using a hypergeometric test (65). The categories are ranked by the value of the sum of all the pairwise multiplication products of the average fold changes.

Overall, only a few gene categories met this criterion. Among the categories that were repressed under limitation conditions, photosynthetic processes are richly represented (PSII, PSI, PBS, and CO_2_ fixation). Two additional processes showing significant change are related to membrane and protein synthesis. The only gene category to exhibit the opposite trend is genes that code for regulators. From this list of functional categories, a subset of differentially regulated genes exhibits significant fold change values under all three conditions. These genes deserve special attention. They are listed in Table S2 and discussed in the following sections. Analysis of their promoter region was conducted, yielding three putative common motifs (Supporting information section: Putative general stress response control and promoter sequences). However, we could not map these putative motifs to known regulatory circuits in *Synechocystis* with confidence.

### PSs and PBSs

**Figure 2:**
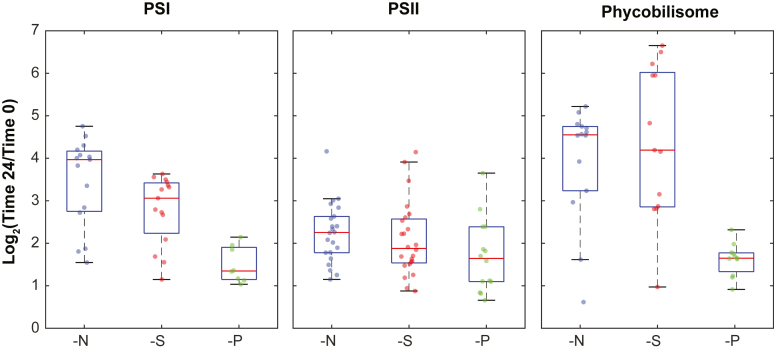
**Transcriptional response of photosynthetic genes.** The plot represents the fold change difference in the transcription of PSI, PSII, and PBS genes (for all genes in the category with *p* values smaller than 0.05, for the given limitation condition). The *boxes* show the interquartile range, with the lower and upper edges corresponding to the 25th and 75th percentiles. The *horizontal line* inside the box represents the median. The “whiskers” extend to the minimum and maximum values within 1.5 times the interquartile range from the quartiles. Individual data points are shown as *dots*. PBS, phycobilisome; PSI, photosystem I; PSII, photosystem II.

For both PS and PBS categories examined, the expression was significantly suppressed under limitation conditions as compared with the recovery for many of the key genes in these complexes (Fig. 2). The extent of repression was similar under N and S limitation and lower upon P limitation. This observation fits well with previous studies on limitation-induced chlorosis (7).

When examining the subset of genes that were significantly repressed under all three limitations, we find many structural genes (Table S2). In PSII, these include oxygen evolution enhancer proteins (PsbO, PsbV, and PsbQ (18, 19)), the cytochrome *b*_559_ genes (PsbF and PsbE), which are considered the scaffold for the assembly of PSII (20), as well as additional small subunits (PsbM/K/I/28 (21, 22)). There is evidence for a role of these proteins on different levels of the assembly of PSII complexes and supercomplexes (23, 24, 25, 26). In PSI, similarly, we observe major structural subunits (PsaA/C/D) as well as more peripheral proteins (PsaK/M) (27). Both PsaD and PsaK were suggested to contribute to the stabilization of PSI complexes (28, 29). In addition, two auxiliary assembly cofactors (Ycf4 and Ycf37) are differentially expressed (30). PBS proteins were the category that exhibited the largest combined fold change under the three conditions (Table 1). The list of genes that were significantly and uniformly affected includes phycocyanin and allophycocyanin proteins as well as rod and core linkers (31, 32). These results fit well with the chlorosis phenomena detected under nutrient deprivation. PBS degradation in cyanobacteria was shown to occur to different extents under N, S, or P limitations (2, 7).

### CO_2_ fixation

During nutrient deprivation, the metabolic pathways alter in reaction to the changing conditions (33). Gene expression results show that indeed under all nutrient depletions tested, functions involved in CO_2_ fixation and the Calvin–Benson cycle are downregulated. One of the strongest and most significant effects, under all three conditions, was on Rubisco (RbcL/S). Additional key Calvin–Benson cycle genes include phosphoribulokinase (34) and further along the cycle, triosephosphate isomerase (35). These proteins are targets for regulation of the cycle activity (36). Considering the reduction in Calvin–Benson cycle enzymes, it is not surprising that carbon-concentrating mechanism genes are also downregulated under nutrient limitations (CcmK/L (37)). With the reduction in light reactions and carbon fixation, it stands to reason that there will be little need for carbon-concentrating mechanism processes (38).

### Protein and membrane biosynthesis

Two categories related to anabolic cellular processes are represented in the list of strongly and uniformly repressed processes (ribosomal proteins, fatty acid, phospholipid, and sterol metabolism, Table 1). The list of uniformly and significantly affected ribosomal genes includes 30 large and small subunit proteins (39). At the top of the affected genes in the lipid category (Table S2) are membrane lipid biosynthesis proteins such as acyl carrier proteins (FabF/D (40)), involved in the elongation of fatty acid chains; SqdB was involved in the glycerolipid sulfoquinovosyl diacylglycerol production (41). The fatty acid desaturase (DesB, Slr1350 (42)) expression was also highly affected by all three limitations. A biotin carboxylation enzyme whose expression level was affected was shown to interact with components of the PII N regulatory system (AccC (43)).

### Regulatory functions

Interestingly, only one category was found to exhibit enhanced expression under all limitation conditions—genes with regulatory functions. The *Synechocystis* 6803 genome includes approximately 50 histidine kinases (Hiks) and about the same number of response regulators (44). They were frequently linked to stress response mechanisms. Taking a closer look into this group of genes, we could identify a subset of 18 genes that were significantly expressed under all three conditions, including Hiks, two-component systems, and pili-associated proteins (Table S1). Many of them were studied in the past and found to be connected to different stress responses. We will survey several interesting examples from that list. Slr2104 is a member of a regulatory two-component system that is connected to cyclic nucleotide signaling cascades (45). Cyclic nucleotide sensors take part in the adaptive response cascade in eukaryotes. Cyanobacteria are the only prokaryotes known to have both cAMP and cGMP (46). Cyclic nucleotide signaling cascades were found to play a role in a variety of acclimation processes (47). Sll1296 was identified as part of the chemotaxis signaling pathway in response to glucose and controlled by Hik8 (48). Motility and chemotaxis are further represented in this group by genes coding for Pili subunits (PilC/G/H (49)). Slr0687 was suggested to be involved in redox signaling (50). The OmpR family kinase sll0789 was identified in studies of cadmium stress (51), salt stress (52), redox regulation, and S starvation (53). In short, the picture that emerges is that of regulators that participate in the response to multiple stress conditions.

### Single gene–level analysis

**Figure 3:**
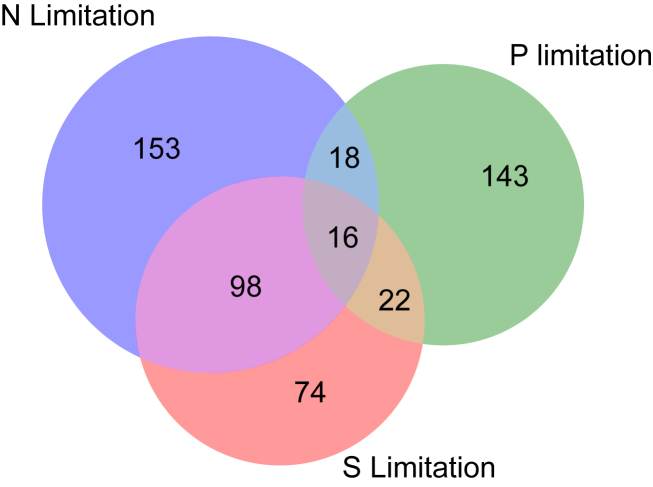
**Highly and consistently expressed genes.** Differentially expressed genes were selected on the basis of the following criteria: log2 fold change greater than 2.5 or lower than −2, *p* values lower than 0.05. The overlap of all three limitation conditions in the Venn diagram includes the list of genes detailed in Table 2.

To complement the category-based analysis, we also evaluated changes on the single gene level. The top-ranking individual genes, differentially expressed under all three conditions (Fig. 3), are reported in Table 2. The list of starvation-repressed transcripts includes several representatives of the chlorophyll biosynthesis pathways (ChlL/N, CPO), ribosomal proteins (L10/12), and a core subunit of PSII (PsbF). The *glgC* gene codes for a glucose phosphorylase participating in glycogen metabolism (54).

**Table 2:** Functional enrichment analysis of differentially expressed genes

ORF	Proposed function	-N	-P	-S	Sum of products
slr0749	ChlL (52)	4.65	5.15	5.35	76.37
slr0750	ChlN (53)	4.9	4.31	5.32	70.13
sll1240	Unknown	3.54	7.25	2.74	55.29
slr1763	Probable methyltransferase	4.38	3.21	3.48	40.44
sll1185	Coproporphyrinogen III oxidase (54)	4.55	2.61	3.77	38.92
sll0253	Hypothetical	4.29	2.6	3.78	37.27
sll1746	50S ribosomal protein L7–L12 dimer	3.16	3.72	3.33	34.67
sll1308	Hypothetical	3.53	2.7	3.93	34.03
sll1745	50S ribosomal protein L10	3.40	3.63	3.02	33.63
ssl3436	Hypothetical	2.55	3.89	3.37	31.66
slr1176	glgC, ADP-glucose pyrophosphorylase (51)	4.1	2.52	3.03	30.39
sll1809	Small ribosomal subunit protein uS8	2.53	3.65	3.14	28.62
ssl3437	Small ribosomal subunit protein uS17	2.50	3.05	3.31	26.01
smr0006	psbF	3.05	2.8	2.87	25.31
slr0271	Unknown	−3.17	−2.99	−2.27	23.42
slr0355	8-Amino-7-xononanoate carboxylating dehydrogenase	−2.48	−3.45	−2.01	20.47

The values represent the average log fold change of gene expression for each deprivation condition. Genes are ranked by the value of the sum of all the pairwise multiplication products of the average fold changes.

### Noncoding RNAs

ncRNAs are major regulators of many cellular processes in cyanobacteria (55). We were expecting to observe ncRNAs, which are differentially expressed. However, none of the ncRNAs described or predicted in the literature exhibited a significant response to all three conditions. In general, N and S limitations shared more ncRNAs with similar expression profiles than those responding to P limitation. Additional discussion of interesting ncRNAs identified in this project can be found in the supplementary information (identification of differentially expressed ncRNAs, Figs. S2 and S3).

### Physiological and biochemical aspects of nutrient limitations

**Figure 4:**
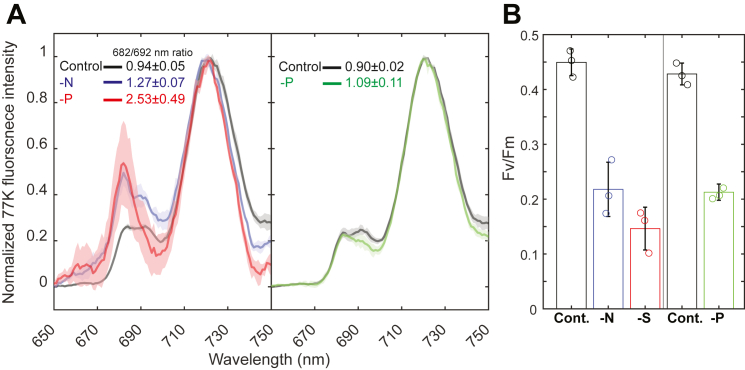
**Chlorophyll fluorescence spectra and the activity of PSII.***A,* low temperature chlorophyll fluorescence (excitation at 430 nm) was measured at the end of the limitation treatment (5 days for nitrogen and sulfur, *left-hand panel*; 8 days for phosphorus, *right-hand panel*). Limited cultures appear in color, whereas the respective control is *black*. Standard deviation (n = 3, biological replicates) appears as a shaded area. The values that appear in the graph are the ratios of 682/692 nm emission intensity. *B,* the maximal potential quantum yield of PSII (Fv/Fm) was measured in limited and control cultures using an imaging PAM, following 5 days of growth for nitrogen and sulfur limitation and 9 days for phosphorus limitation. Fm values were measured in the presence of the PSII inhibitor DCMU. Error bars represent standard deviation (n = 3, biological replicates). DCMU, 3-(3,4-dichlorophenyl)-1,1-dimethylurea; PAM, pulse-amplitude modulated; PSII, photosystem II.

Considering the prominence of chlorophyll biosynthesis and photosynthetic complexes in the transcriptomic results, we further validated and investigated these processes on the protein and physiological levels. In these experiments, we followed the progression of N, P, or S limitation (Fig. 1*A*). We observed a growth arrest under all three conditions. As discussed previously, the extent and rate of reduction in growth, as compared with control cultures, were different for the different treatments. Interestingly, cell counts were affected to a greater degree than absorbance values, which may indicate a difference in cell sizes, a parameter that absorbance at 730 nm measurements is sensitive to. While for all deprivation conditions, growth arrest requires several days, the increase in chlorophyll concentrations was stopped immediately and remained constant from the start, in limited cultures. Therefore, cells divided but did not produce additional chlorophyll (Fig. 1*A*). These results correspond well with the transcriptional profiles (Tables 1 and 2). Most of the chlorophyll in cyanobacteria is associated with PSI. PSI contains more chlorophylls and is present in cyanobacterial cells at 5/1 to 10/1 excess over PSII (56). The amount of photo-oxidizable PSI in the culture was monitored as the kinetic absorption change under illumination in the presence of 3-(3,4-dichlorophenyl)-1,1-dimethylurea and 2,5-dibromo-3-methyl-6-isopropylbenzoquinone, blocking electron transport to PSI (Fig. 1*A*: ΔAmax values (14)). These values followed the chlorophyll concentration values closely (the *R*^2^ value for a linear fit of all the data presented in Fig. 1 for chlorophyll concentration *versus* ΔAmax is 0.86), indicating that PSI activity is limited to a similar degree under all three limitation conditions. Additional information on the composition of the photosynthetic apparatus was collected through 77K chlorophyll fluorescence emission spectroscopy (Fig. 4*A*). It is important to note that these values are relative and are presented normalized to the highest peak (PSI peak at ∼720 nm). As compared with their respective control curves, significant differences are observed under all three limitation conditions at the 670 to 700 nm range. PSII peaks are found in this range (∼685 and ∼692 nm, see control curves). However, a shorter wavelength peak, at ∼682 nm, is observed under all three limitation conditions to a different degree (Fig. 4*A*). Furthermore, the PSI peak in limited cultures is blue shifted as compared with control cultures. The 682 nm spectral region is known to represent isiA fluorescence. However, this protein is induced strictly under Fe limitation (12) and was not found to be differentially expressed in our dataset. Therefore, these shorter wavelength peaks are most probably the result of degradation/disassembly products of PSI, PSII, or both (as suggested by Keren *et al.* (57)). To gain more information on PSII, we measured its activity by pulse-amplitude modulated fluorimetry (Fig. 2*B*). The maximal potential quantum yield of PSII was lower under all limiting conditions, consistent with the increase in the peak at 675 nm. Taken together with the results in Figure 3, these results indicate a reduced content of active PS, mainly PSI, under limited conditions.

**Figure 5:**
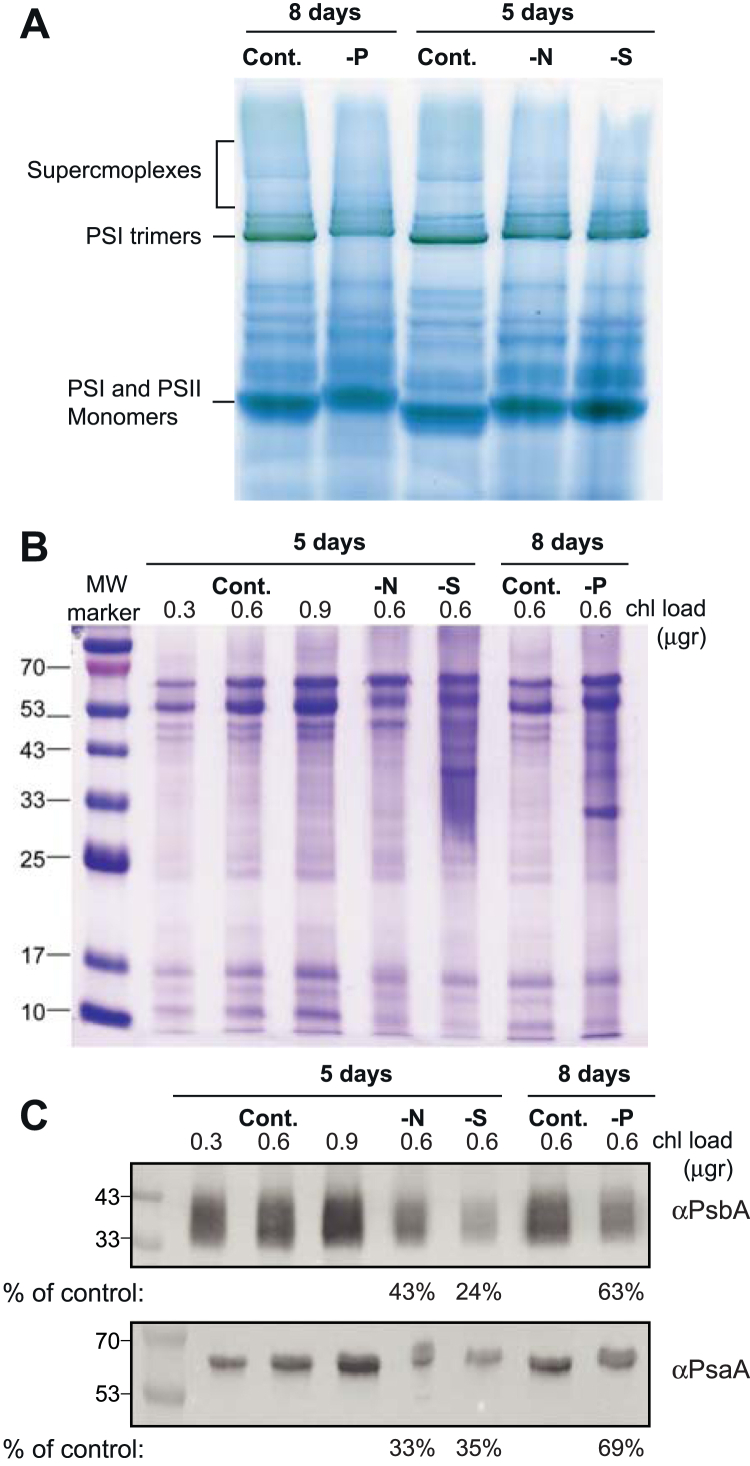
**BN gel and Western blot analysis.** Cultures were harvested and processed following 5 (-N, -S) or 8 days (-P) of growth under nutrient limitation. *A,* BN gel, the position of photosynthetic complexes is indicated on the *right-hand side*, based on experience from previous studies (62). Gels were loaded on an equal chlorophyll basis. (*B*) Coomassie stained SDS-PAGE gel and (*C*) Western blot analysis. Chlorophyll loads are indicated at the *top* of the lane. Semiquantitative analysis of band intensity was performed using ImageJ. The results are presented as a percent of the control. BN, blue native.

Nutrient limitation–induced changes in the composition of the photosynthetic apparatus are also evident in blue native (BN)-gel analysis of pigmented protein complexes (Fig. 5*A*). In all three limitation treatments, a reduction in PSI trimer and photosynthetic supercomplexes was observed. PSI and PSII monomer bands were of similar intensity, but control monomer bands migrated to a longer distance in the gel. The content of the major PS components, PsaA and PsbA, was also reduced as compared with control conditions, on an equal chlorophyll basis (Fig. 5, *B* and C). These results, collectively, support a change in the organization of photosynthetic complexes in the thylakoid membranes. The changes exhibit a very similar profile under all three limitation conditions.

## Discussion

In this study, we aimed to identify the shared molecular and physiological basis underlying cyanobacterial responses to nutrient limitation. By integrating transcriptomic profiles with physiological data, we identified a core set of conserved processes activated under N, P, and S limitation. Among the most prominent and consistent responses was the modulation of tetrapyrrole biosynthesis and pigment-binding proteins, underscoring the centrality of photosynthetic pigment regulation in nutrient stress acclimation.

A particularly striking observation was the downregulation of genes involved in chlorophyll *a* biosynthesis, especially those encoding components of the dark-operative protochlorophyllide reductase (DPOR) complex—*chlL*, *chlN*, and *chlB*. DPOR catalyzes chlorophyll *a* synthesis in the absence of light, and its expression was significantly suppressed across all tested conditions. This suggests a generalized suppression of chlorophyll biosynthesis under nutrient stress, including both the light-independent and, to a lesser extent, the light-dependent pathways. These findings are in line with recent work implicating DPOR components as key factors in the recovery from N starvation in dormant and chlorotic cyanobacteria (58).

The regulation of light-independent chlorophyll biosynthesis genes, such as *chlL*, *chlN*, and *chlB*, is particularly meaningful in natural environments, where cyanobacteria often experience diurnal light cycles and fluctuating conditions. In such contexts, the ability to synthesize chlorophyll *a* in the dark can be critical for survival and recovery during night-time or light-limited periods. However, under standard laboratory conditions—where cultures are typically grown under continuous white light—this regulatory response may appear less consequential. As a result, the physiological impact of DPOR suppression may be underestimated in artificial settings. Previous studies have shown that the expression of *chlL/N* is tightly linked to dark–light transitions and redox status (58, 59), supporting its key role in fluctuating environments.

Physiologically, this effect was evident from the immediate arrest of chlorophyll accumulation following the onset of starvation (Fig. 1*A*). These changes were closely associated with the downregulation of genes encoding structural components and assembly cofactors of PSI and PSII, as well as PBSs, resulting in reduced PSI activity, PSII quantum yield, and altered organization of PS complexes and supercomplexes (Figs. 4 and 5). Concomitantly, transcripts associated with protein and membrane biosynthesis were also suppressed (Table 1), reflecting a broader metabolic slowdown.

Importantly, our analysis revealed that a limited subset of kinases and two-component regulatory systems were consistently upregulated under all nutrient limitation conditions. This suggests the existence of a conserved signaling network that coordinates the cellular response to diverse nutrient stresses.

Collectively, our results support the existence of a core regulatory and functional module that governs cyanobacterial acclimation to macronutrient stress. However, many of the transcriptional and physiological responses, observed here, are not unique to specific nutrient limitation(s). For example, regulatory gene expression, disassembly of photosynthetic complexes, and changes in supercomplex architecture have also been reported in studies of other stress conditions. In a previous study, we observed PSI trimer monomerization and complex disassembly under Mn limitation (13), which we initially interpreted as an Mn-specific adaptation. However, our current findings prompt a re-evaluation of that interpretation, suggesting that these are more likely general stress responses. Similarly, chlorophyll biosynthesis is also impaired under Fe limitation, whereas other stress-specific responses—such as *isiAB* induction (Fe limitation) or flavodoxin accumulation (Cu limitation)—highlight the need to differentiate between general and specific elements of stress responses (60). This distinction extends to ncRNAs and kinases, where several of the regulatory genes identified here have been previously implicated in broader stress response pathways (61, 62, 63, 64).

In conclusion, our study uncovers a common foundation for nutrient limitation responses. These findings raise the possibility that a hierarchical organization underlies these responses. Based on our data, we propose a model in which the suppression of chlorophyll biosynthesis, the recycling of stalled ribosomes *via* the ssrA-SmpB system, and the degradation of nonessential or incomplete proteins form a central regulatory axis. Although this study does not definitively identify the regulatory network governing the shared response, it does highlight putative regulatory sequences and a subset of Hiks and response regulators as candidate components of this regulatory circuit.

## Experimental procedures

### Growth conditions

Stock cultures of *Synechocystis sp*. strain PCC 6803 were grown in YBG11 (60). Cultures were maintained at 30 °C with constant shaking under a light intensity of 50 μmol photons m^-2^s^-1^. For nutrient limitation experiments, cultures were transferred to -N, -S, or -P depleted YBG11. To achieve nutrient depletion, cells were centrifuged, resuspended in the appropriate depleted medium, and washed three times with the same medium before being adjusted to an absorbance of 0.1 at 730 nm. Control cultures underwent the same treatment but were resuspended in complete YBG11. For recovery experiments, after 5 days of N or S depletion, or 8 to 10 days of P depletion, cultures were centrifuged at 3500*g* for 10 min and resuspended in complete YBG11. The cultures were then allowed to grow for an additional 72 h.

### Spectroscopy and microscopy

Chlorophyll concentrations were measured in samples extracted in 100% methanol. Absorbance was measured using a Shimadzu UV-1900i spectrophotometer at 665 nm (61). The absorbance was measured at 730 nm. Cell growth was monitored by hemocytometer cell counts. Chlorophyll fluorescence spectra (77 K) were measured using a PTI Quantamaster spectrofluorometer (14). PSI activity was measured as P700 photo-oxidation using the Joliot-type spectrophotometer. In order to block electron flow to PSI completely, 10 Μm 3-(3,4-dichlorophenyl)-1,1-dimethylurea and 10 Μm 2,5-dibromo-3-methyl-6-isopropylbenzoquinone were added (14).

### Membrane isolation and BN gels

Membrane extractions and BN-PAGE were performed (62, 63). Following starvation, 1 l of cells was harvested by centrifugation at 3500*g* for 10 min. The cells were then washed with washing buffer (50 mM Hepes, pH 7.5, 30 mM CaCl_2_) and resuspended in breaking buffer (50 mM Hepes, pH 7.5, 30 mM CaCl_2_, 800 mM sorbitol, 1 mM aminocaproic acid, and an antiprotease cocktail). Cell lysis was performed using a Constant System Cell Disruptor (CF1) at 30,000 psi. The lysate was centrifuged at 3000*g* for 5 min, and the supernatant was collected and further centrifuged at 17,000*g* for 20 min. The resulting pellet, containing the thylakoids, was stored at −80 °C until use. All steps were carried out at 4 °C. BN-PAGE was performed as follows: Dodecyl maltoside (4%) was added at a 1:1 ratio to a final concentration of 0.5 mg chlorophyll/ml in resuspension buffer (20% glycerol, 25 mM Bis–Tris, 10 mM MgCl_2_, pH 7). Samples were incubated on ice for 30 min, followed by centrifugation at 18,000*g* for 20 min at 4 °C. The supernatant was transferred to a new test tube, and 5% Serva Blue solution (5% Serva Blue, 100 mM Bis–Tris, pH 7, 30% sucrose, 500 mM aminocaproic acid, and 10 mM EDTA) was added at one-tenth of the sample volume. Samples were separated on native 4.5% to 12.5% acrylamide gels and run for approximately 4 h.

### RNA extraction

RNA extraction was conducted according to Wang *et al.* (64). Cells were harvested by centrifugation at 3500*g* for 10 min. The pellets were then resuspended in PGTX (phenol, guanidinium, thiocyanate, and xenon) and incubated at 65 °C in a water bath for 15 min. After adding 700 μl of chloroform/isoamyl alcohol (24:1) and thorough mixing, samples were incubated at room temperature for 10 min. They were then centrifuged using a fixed-angle rotor at 3480*g* at 23 °C for 5 min. The upper aqueous phase was transferred to a new vial, and an equal volume of chloroform/isoamyl alcohol (24:1) was added and mixed. After a second centrifugation under the same conditions, the aqueous phase was transferred again and mixed with an equal volume of isopropanol. The tubes were gently inverted to facilitate mixing, and RNA was precipitated overnight at −20 °C. RNA was pelleted by centrifugation at 14,500*g*, 4 °C, for 30 min. The pellet was washed with 200 μl of 70% ethanol and centrifuged again at 14,500*g*, 4 °C, for 10 min. After air drying for 10 min, the pellet was resuspended in 20 μl of H_2_O. RNA-Seq analysis was performed in the Life Sciences sequencing facility of the Hebrew University.

### RNA-Seq data processing and analysis

Raw RNA-Seq data were processed using the Galaxy platform. Adapter sequences and low-quality reads were trimmed with Trimmomatic. Cleaned reads were aligned to the *Synechocystis* sp. PCC 6803 reference genome using HISAT2. Gene expression was quantified with featureCounts based on the corresponding genome annotation.

Differential expression analysis was carried out using DESeq2, which models count data to identify significantly regulated genes. Genes with an adjusted *p* value (Benjamini–Hochberg) below 0.05 were considered differentially expressed.

Functional enrichment analysis was performed using a custom R script adapted from published code. Enrichment of functional categories was assessed using a hypergeometric test. Only categories with enrichment *p* values <0.05 were retained.

Putative ncRNAs were identified using Rockhopper, a tool optimized for bacterial transcriptomics. Reads were aligned using Rockhopper's internal aligner, and transcript prediction was based on coverage outside annotated coding sequences. Candidate ncRNAs were classified based on their genomic position, expression levels, and the absence of overlap with protein-coding genes. These were manually curated and analyzed for differential expression under nutrient stress conditions.

### Data availability

Physiological and biochemical data are available upon request. The transcriptomic data were uploaded to the National Center for Biotechnology Information repository (Submission ID: SUB15573133, BioProject ID: PRJNA1311732).

## Supporting information

This article contains supporting information. (18, 19, 20, 21, 22, 27, 30, 31, 32, 35, 37, 39, 40, 42, 43, 45, 48, 49, 50, 66, 67, 68, 69, 70, 71, 72, 73, 74, 75, 76)

## Conflict of interest

The authors declare that they have no conflicts of interest with the contents of this article.
