# Supplementary material for: A shared basis for nutrient limitation response in cyanobacteria
Source: J Biol Chem. 2025 Sep 24;301(11):110765. doi: 10.1016/j.jbc.2025.110765 (PMC12589917; doi:10.1016/j.jbc.2025.110765)
Supplement: Supporting information [file mmc1.docx]

**A shared basis for nutrient limitation response in cyanobacteria**

^1^Hagit Zer, ^1^Stav Chen, ^1^David Rasin, ^2^Miguel Hernandez – Prieto, ^1*^Nir Keren

^1^Institute of Life Sciences. The Hebrew University of Jerusalem. ^2^School of Chemical engineering, University of New South Wales.

*Corresponding author: Nir Keren ([nir.ke@mail.huji.ac.il](mailto:nir.ke@mail.huji.ac.il))

**Supplemental material**

**Supplemental table 1: top expressed genes under limitation conditions**

| *Transcription Start* | *Translation Start* | *Translation Stop* | *Transcription Stop* | *Strand* | *Name* | *Synonym* | *Product* | *Expression 0N* | *Expression 0S* | *Expression 0P* |
| --- | --- | --- | --- | --- | --- | --- | --- | --- | --- | --- |
| 3315962 |  |  | 3315536 | - | ssrA | SYNGTS_RS16565 | RNA molecule itself, also called tmRNA or 10Sa RNA | 370595 | 492525 | 194166 |
| 153057 |  |  | 153655 | + | rnpB | SYNGTS_nc0001 | - | 50084 | 71684 | 58434 |
| 1996634 | 1996607 | 1996314 | 1996234 | - | ssl1762 | SYNGTS_1819 | Probably related to CO2 assimilation | 28376 | 5978 | 7017 |
| 2375878 | 2375995 | 2376345 | 2376395 | + | slr0376 | SYNGTS_2132 | In an operon related to CO2 assimilation | 23566 | 13295 | 9205 |
| 2789871 | 2789891 | 2790343 | 2790525 | + | slr0915 | SYNGTS_2501 | putative endonuclease | 20772 | 7972 | 21478 |
| 3180922 | 3180830 | 3180525 | 3180423 | - | rbpA | SYNGTS_2848 | RNA binding protein | 14911 | 6318 | 2759 |
| 1818867 | 1818814 | 1817732 | 1817683 | - | psbA3 | SYNGTS_1655 | photosystem II D1 protein | 14878 | 12431 | 7595 |
| 1116757 | 1116629 | 1116360 | 1116301 | - | ssl2501 | SYNGTS_1011 | PHA surface-coating protein (phasin), PhaP | 13146 | 2348 | 11131 |
| 7225 | 7229 | 8311 | 8358 | + | psbA2 | SYNGTS_0008 | photosystem II D1 protein | 12113 | 11636 | 7164 |
| 2373886 | 2373886 | 2374266 | 2374266 | + | slr0373 | SYNGTS_2130 | In an operon related to CO2 assimilation | 8278 | 16053 | 1877 |
| 3180422 |  |  | 3180397 | - | - | asRbpA | antisense: AsRNA of RbpA involved in cold stress adaptation | 7497 | 3720 | 1925 |
| 2374267 | 2374357 | 2375862 | 2375867 | + | slr0374 | SYNGTS_2131 | cell division cycle protein | 7209 | 17630 | 2941 |
| 3228538 | 3228597 | 3229655 | 3229734 | + | psbD2 | SYNGTS_2888 | photosystem II D2 protein | 5589 | 2356 | 2408 |
| 302831 | 303424 | 303621 | 303621 | + | ssr2153 | SYNGTS_0277 | Probably related to CO2 assimilation | 5340 | 6818 | 18277 |
| 3283740 | 3283662 | 3283099 | 3283093 | - | sll1338 | SYNGTS_2936 | hypothetical protein | 5339 | 6533 | 6146 |
| 1135305 | 1135337 | 1135588 | 1135596 | + | ssr1480 | SYNGTS_1023 | RNA-binding protein | 4727 | 1695 | 3001 |
| 2525087 | 2525023 | 2524643 | 2524443 | - | petE | SYNGTS_2263 | plastocyanin | 4324 | 4019 | 5097 |
| 3065026 | 3065024 | 3064434 | 3064434 | - | sll0788 | SYNGTS_2747 | hypothetical protein | 3530 | 4326 | 1338 |
| 1352734 | 1352721 | 1352413 | 1352301 | - | sll0846 | SYNGTS_1228 | hypothetical protein | 3465 | 3414 | 5151 |
| 1694555 |  |  | 1694329 | - | - | asCruF | antisense of CruF a carotenoid 1,2-hydratase enzyme | 3358 | 1487 | 3455 |
| 460695 | 460690 | 460250 | 460148 | - | hsp17 | SYNGTS_0420 | 16.6 kDa small heat shock protein, molecular chaperon | 3312 | 1577 | 1425 |
| 2694426 | 2694437 | 2694592 | 2694696 | + | ssr0692 | SYNGTS_2424 | regulates flux into the ornithine-ammonia cycle (OAC), the key hub of cyanobacte | 2696 | 7706 | 9452 |
| 1296316 | 1296316 | 1295810 | 1295810 | - | hofG | SYNGTS_1175 | general secretion pathway protein G | 2505 | 1961 | 3493 |
| 1241806 | 1241806 | 1241582 | 1241491 | - | ssl3364 | SYNGTS_1123 | hypothetical protein | 2334 | 3002 | 1388 |
| 944187 | 944187 | 946382 | 946423 | + | psaB | SYNGTS_0864 | photosystem I P700 chlorophyll a apoprotein A2 | 2260 | 1633 | 9043 |
| 1677840 |  |  | 1678212 | + | - | ncRNA_1677840 | 5'UTR of transposase | 2230 | 1096 | 3845 |
| 1885755 |  |  | 1886129 | + | ncRNA ssaA | predicted RNA | antisense: sll1166 ssrR | 2156 | 1267 | 1764 |
| 1426666 | 1426468 | 1421243 | 1421173 | - | sll1951 | SYNGTS_1295 | hemolysin | 2089 | 1576 | 2779 |
| 2313390 | 2313390 | 2311480 | 2311407 | - | dnaK | SYNGTS_2096 | DnaK protein | 2063 | 3040 | 4585 |
| 2450537 |  |  | 2447655 | - | rrn23Sa | SYNGTS_r0002 | 23S ribosomal RNA | 2044 | 2632 | 1591 |
| 3325823 |  |  | 3328705 | + | rrn23Sb | SYNGTS_r0005 | 23S ribosomal RNA | 2039 | 2636 | 1582 |
| 1953538 | 1953538 | 1953888 | 1953986 | + | slr1674 | SYNGTS_1781 | PSII thermotolerance/slr1674 encodes a protein which stimulates maturation of | 1969 | 1871 | 1704 |
| 3328778 |  |  | 3328896 | + | rrn5Sb | SYNGTS_r0006 | 5S ribosomal RNA | 1930 | 1163 | 1222 |
| 941677 | 941686 | 943941 | 944186 | + | psaA | SYNGTS_0863 | photosystem I P700 chlorophyll a apoprotein A1 | 1882 | 1823 | 7219 |
| 2447582 |  |  | 2447464 | - | rrn5Sa | SYNGTS_r0001 | 5S ribosomal RNA | 1875 | 1196 | 1217 |
|  | 1347673 | 1346291 | 1346223 | - | psbC | SYNGTS_1223 | photosystem II CP43 protein | 1844 | 1300 | 2624 |
| 2269140 | 2268979 | 2267942 | 2267889 | - | rpoD | SYNGTS_2057 | RNA polymerase sigma factor | 1776 | 1997 | 1238 |
| 2467688 | 2467688 | 2465223 | 2465197 | - | clpC | SYNGTS_2217 | ATP-dependent Clp protease regulatory subunit | 1646 | 1752 | 1529 |
| 303622 |  |  | 303799 | + | ssr2153-ncRNA | predicted RNA | Targets related to translation | 1546 | 2382 | 4788 |
| 2822761 |  |  | 2822505 | - | - | asSlr0580 | antisense: SYNGTS_2539; aminotransferase class I/II-fold pyridoxal phosphate- | 1467 | 1522 | 1934 |
| 1348911 | 1348715 | 1347657 |  | - | psbD | SYNGTS_1224 | photosystem II D2 protein | 1347 | 884 | 1788 |
| 2452491 |  |  | 2451003 | - | rrn16Sa | SYNGTS_r0003 | 16S ribosomal RNA | 1252 | 1345 | 973 |
| 3323869 |  |  | 3325357 | + | rrn16Sb | SYNGTS_r0004 | 16S ribosomal RNA | 1245 | 1346 | 970 |
| 1606170 | 1606170 | 1606769 | 1606878 | + | sodB | SYNGTS_1451 | superoxide dismutase | 1115 | 1643 | 2260 |
| 92583 |  |  | 93098 | + | - | predicted RNA | Same level | 1088 | 1102 | 1131 |
| 811257 | 811245 | 810922 | 810593 | - | sml0011 | SYNGTS_0729 | hypothetical protein | 1070 | 1072 | 1450 |

This list is based on the 100 top expressed genes for each of the limitation conditions. The list includes a subset of 48 genes found to be common to the three conditions.


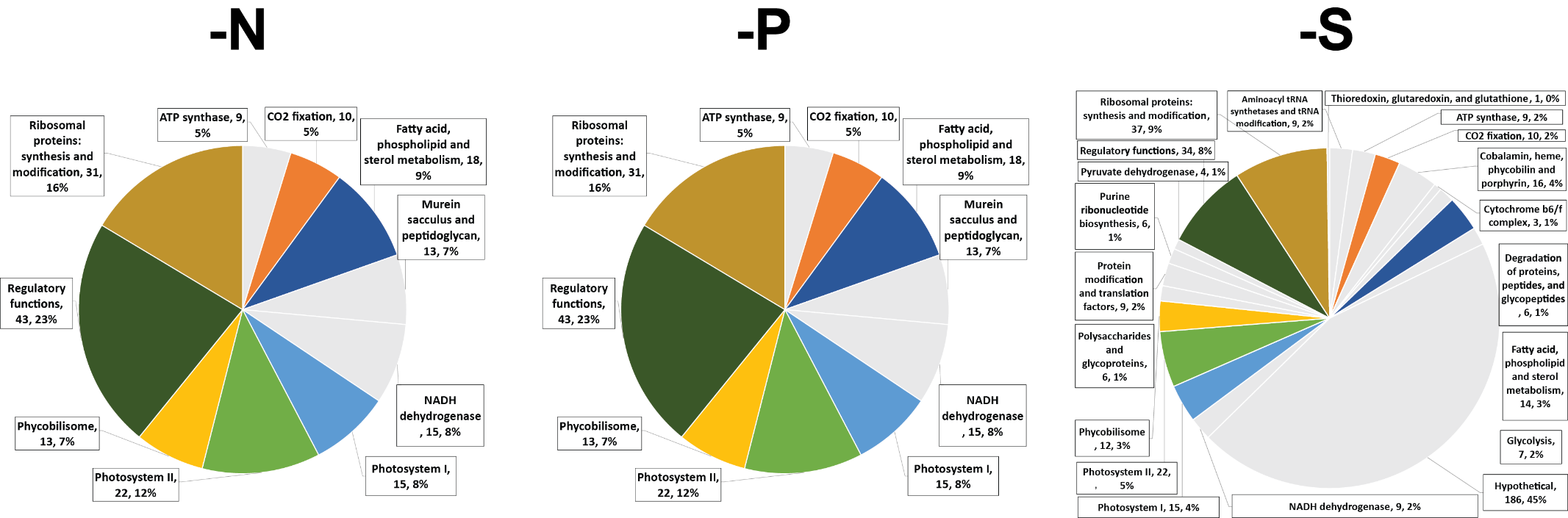


**Supplemental Figure 1: Differentially enriched gene categories in each of the conditions.**

Pie charts presenting data for gene categories that were filtered using a p-adjusted value threshold of 0.05 per gene and a p-value threshold of 0.05 per category. Only categories with an average log-fold-change values greater than 1 or less than -1 were included. Functional enrichment was assessed using a hypergeometric test. The coloured slices are the categories that overlap among all the starvation conditions (as in Table 1). The uncoloured (gray) slices represent categories that do not meet this criterion. The description of each slice is composed of the name of the category, the number of genes that are differentially expressed, and their percentage from the total genes in the category.

**Supplemental Table 2: Differentially expressed gene under all conditions within enriched categories.**

| **category** | **Name** | **-N** | **-P** | **-S** | **Sum of products** | **Suggested function** |
| --- | --- | --- | --- | --- | --- | --- |
| **CO_2_ fixation** | | | | | | |
| slr0012 | rbcS | 4.47 | 2.43 | 4.15 | 39.54 | ribulose bisphosphate carboxylase small subunit |
| slr0009 | rbcL | 4.63 | 1.97 | 4.11 | 36.22 | Ribulose bisphosphate carboxylase large subunit |
| sll1030 | ccmL | 3.07 | 2.07 | 3.48 | 24.21 | carbon dioxide-concentrating mechanism protein (1) |
| sll1029 | ccmK | 2.98 | 2.26 | 3.28 | 23.91 | carbon dioxide-concentrating mechanism protein (1) |
| sll1028 | ccmK | 3.14 | 2.03 | 3.39 | 23.90 | carbon dioxide-concentrating mechanism protein (1) |
| sll1525 | prk | 3.70 | 1.49 | 2.28 | 17.35 | Phosphoribulokinase (2) |
| sll1342 | gap2 | 2.39 | 2.34 | 1.91 | 14.62 | glyceraldehyde-3-phosphate dehydrogenase (3) |
| slr0783 | tpi | 1.49 | 1.43 | 1.76 | 7.27 | triosephosphate isomerase (4) |
| **Fatty acid, phospholipid and sterol metabolism** | | | | | | |
| sll0262 | desD | 2.48 | 2.59 | 2.65 | 19.84 | delta-6 desaturase (5) |
| ssl2084 | acpP | 2.31 | 2.45 | 2.33 | 16.72 | acyl carrier protein (6) |
| slr1350 | Slr1350 | 2.98 | 2.28 | 1.31 | 13.65 | acyl-lipid desaturase (5) |
| sll1069 | fabF | 2.37 | 1.89 | 2.10 | 13.42 | beta ketoacyl-acyl carrier protein synthase (6) |
| slr1020 | sqdB | 2.51 | 1.14 | 2.48 | 11.92 | 3-oxoacyl-[acyl-carrier protein] reductase (6) |
| slr2023 | fabD | 2.81 | 1.86 | 1.43 | 11.86 | malonyl coenzyme A-acyl carrier protein transacylase (6) |
| sll0053 | accC | 2.34 | 1.55 | 1.53 | 9.60 | biotin carboxylase (7) |
| slr1051 | fabI | 1.77 | 1.81 | 1.64 | 9.10 | enoyl-[acyl-carrier-protein] reductase (6) |
| **Photosystem I** | | | | | | |
| slr0737 | psaD | 4.20 | 1.96 | 3.36 | 28.91 | Photosystem I reaction center subunit II (8) |
| smr0005 | psaM | 3.83 | 1.85 | 3.50 | 26.96 | Photosystem I reaction center subunit XII (8) |
| slr1834 | psaA | 4.75 | 1.03 | 3.56 | 25.52 | Photosystem I P700 chlorophyll a apoprotein (8) |
| sml0008 | psaA | 4.00 | 1.36 | 3.44 | 23.89 | Photosystem I reaction center subunit IX (8) |
| ssl0563 | psaC | 4.30 | 1.13 | 3.27 | 22.59 | Photosystem I iron-sulfur center (8) |
| sll0629 | psaK2 | 2.84 | 1.17 | 2.09 | 11.69 | Photosystem I reaction center subunit PsaK (8) |
| slr0171 | ycf37 | 1.87 | 2.14 | 1.55 | 10.25 | Photosystem I auxiliary assembly factor (9) |
| sll0226 | ycf4 | 1.81 | 1.34 | 1.69 | 7.72 | Photosystem I auxiliary assembly factor (9) |
| **Photosystem II** | | | | | | |
| sml0001 | psbI | 2.63 | 2.39 | 4.15 | 27.09 | Photosystem II reaction center protein I (10) |
| smr0006 | psbF | 3.05 | 2.80 | 2.87 | 25.31 | Cytochrome *b*_559_ subunit beta (11) |
| sll0427 | psbO | 4.16 | 1.10 | 3.91 | 25.15 | Photosystem II extrinsic protein O (12) |
| ssr3451 | psbE | 2.84 | 2.39 | 2.53 | 20.05 | Cytochrome *b*_559_ subunit alpha (11) |
| sll1638 | psbQ | 2.58 | 1.82 | 3.47 | 19.95 | Cyano Q (13) |
| sll0258 | psbV | 3.01 | 1.70 | 2.69 | 17.78 | Photosystem II extrinsic protein V (12) |
| sll1398 | psb28 | 1.15 | 3.65 | 1.58 | 11.77 | Photosystem II reaction center Psb28 protein (14) |
| sml0003 | psbM | 2.40 | 1.59 | 1.91 | 11.43 | Photosystem II reaction center protein M (10) |
| sml0005 | psbK | 1.78 | 1.10 | 1.96 | 7.62 | Photosystem II reaction center protein K (10) |
| **Phycobilisome** (15, 16) | | | | | | |
| sll1577 | cpcB | 4.54 | 1.78 | 6.65 | 50.10 | phycocyanin β subunit subunit |
| sll1578 | cpcA | 4.57 | 1.69 | 6.50 | 48.37 | phycocyanin α subunit subunit |
| sll1579 | cpcC2 | 4.80 | 1.64 | 6.22 | 47.94 | phycobilisome rod linker polypeptide CpcC2 subunit |
| sll1580 | cpcC1 | 4.75 | 1.75 | 5.95 | 47.01 | phycobilisome linker polypeptide CpcC1 subunit |
| ssl3093 | cpcD | 4.71 | 1.65 | 5.95 | 45.63 | phycobilisome small rod linker polypeptide CpcD |
| ssr3383 | apcC | 5.08 | 1.98 | 4.83 | 44.13 | phycobilisome small core linker polypeptide ApcC |
| slr1986 | apcB | 4.54 | 1.63 | 4.19 | 33.22 | Allophycocyanin beta chain |
| slr2067 | apcA | 4.61 | 1.23 | 4.16 | 29.99 | Allophycocyanin alpha chain |
| slr1459 | apcF | 2.97 | 2.32 | 2.87 | 22.04 | Allophycocyanin subunit beta-18 |
| slr0335 | apcE | 3.92 | 0.91 | 3.15 | 18.83 | Phycobiliprotein ApcE |
| **Regulatory functions** | | | | | | |
| slr2104 | Slr2104 | -1.96 | -2.34 | -1.93 | 12.89 | two-component regulatory system (17) |
| sll1296 | CheA like | -1.13 | -3.85 | -1.19 | 10.28 | two-component hybrid sensor and regulator (18) |
| slr0687 | Slr0687 | -2.11 | -1.80 | -1.53 | 9.77 | Redox signaling (19) |
| slr1400 | hik38 | -2.43 | -1.38 | -1.45 | 8.88 |  |
| sll0789 | copR | -1.87 | -1.17 | -2.16 | 8.78 | OmpR subfamily (20) |
| sll1905 | Hik19 | -1.24 | -2.25 | -1.55 | 8.20 | control cold-inducible expression of the desB promoter |
| slr0533 | hik10 | -1.64 | -1.33 | -1.96 | 7.97 | histidine kinase |
| slr2098 | Hik21 | -0.91 | -1.56 | -1.45 | 4.97 | histidine kinase |
| slr1324 | Slr1324 | -1.21 | -1.67 | -0.78 | 4.27 | Two-component hybrid sensor and regulator |
| sll1392 | pfsR | -0.96 | -1.21 | -1.42 | 4.26 | represses the expression of bfr genes under low light conditions |
| sll1334 | Sll1334 | -0.94 | -1.33 | -1.25 | 4.08 | putative histidine kinasse |
| slr1285 | Hik34 | -1.30 | -1.29 | -0.89 | 3.99 | the regulation of thermotolerance |
| sll1228 | Hik4 | -0.79 | -1.11 | -1.40 | 3.54 | two-component hybrid sensor and regulator |
| slr1225 | spkF | -1.06 | -1.21 | -0.91 | 3.36 | Serine/threonine-protein kinase |
| sll0396 | Sll0396 | -1.01 | -0.72 | -1.13 | 2.68 | OmpR subfamily |
| slr0322 | pilLC | -0.88 | -1.11 | -0.82 | 2.60 | CheA-like protein (21) |
| slr1041 | pilG | -0.56 | -1.09 | -1.09 | 2.40 | Motility and natural transformation competency (21) |
| slr1042 | pilH | -1.09 | -0.80 | -0.79 | 2.36 | CheY subfamily \ involved in the pilus biogenesis (21) |
| sll1624 | Sll1624 | -0.75 | -0.74 | -1.14 | 2.25 | Regulatory components of sensory transduction system |
| sll1387 | pppA | -0.62 | -0.88 | -1.09 | 2.17 | serine/threonine protein phosphatase PppA nitrogen starvation |
| slr1697 | spkB | -0.66 | -1.35 | -0.53 | 1.96 | Serine/threonine-protein kinase B [2] |
| **Ribosomal proteins: synthesis and modification**(22) | | | | | | |
| sll1746 | rplL | 3.16 | 3.72 | 3.34 | 34.67 | 50S ribosomal subunit protein L7/L12 dimer |
| sll1745 | rplJ | 3.40 | 3.63 | 3.02 | 33.63 | 50S ribosomal protein L10 |
| ssl3436 | rpmC | 2.55 | 3.89 | 3.37 | 31.66 | Large ribosomal subunit protein uL29 |
| sll1809 | rpsH | 2.53 | 3.65 | 3.14 | 28.62 | Small ribosomal subunit protein uS8 |
| sll1808 | rplE | 2.36 | 3.45 | 3.08 | 26.02 | Large ribosomal subunit protein uL5 |
| ssl3437 | rpsQ | 2.50 | 3.05 | 3.31 | 26.01 | Small ribosomal subunit protein uS17 |
| sll1805 | rplP | 2.42 | 3.19 | 2.95 | 24.23 | Large ribosomal subunit protein uL16 |
| ssr1399 | rpsR | 1.61 | 5.16 | 2.33 | 24.07 | Small ribosomal subunit protein bS18 |
| sll1806 | rplN | 2.42 | 3.09 | 2.95 | 23.68 | Large ribosomal subunit protein uL14 |
| sll1812 | rpsE | 2.37 | 2.65 | 3.06 | 21.63 | Small ribosomal subunit protein uS5 |
| sll1801 | rplW | 2.57 | 2.94 | 2.39 | 20.72 | Large ribosomal subunit protein uL23 |
| sll1810 | rplF | 2.36 | 2.71 | 2.70 | 20.06 | Large ribosomal subunit protein uL6 |
| sll1799 | rplC | 2.29 | 3.03 | 2.37 | 19.49 | Large ribosomal subunit protein uL3 |
| sll1813 | rplO | 2.34 | 2.41 | 2.74 | 18.67 | Large ribosomal subunit protein uL15 |
| sll1804 | rpsC | 2.37 | 2.47 | 2.58 | 18.35 | Small ribosomal subunit protein uS3 |
| sll1802 | rplB | 2.47 | 2.64 | 2.32 | 18.35 | Large ribosomal subunit protein uL2 |
| sll1811 | rplR | 2.13 | 2.48 | 2.82 | 18.32 | Large ribosomal subunit protein uL18 |
| ssl3432 | rpsS | 2.36 | 2.38 | 2.52 | 17.57 | Small ribosomal subunit protein uS19 |
| sll1800 | rplD | 2.35 | 2.50 | 2.24 | 16.75 | Large ribosomal subunit protein uL4 |
| sll1744 | rplA | 2.41 | 2.18 | 2.42 | 16.35 | Large ribosomal subunit protein uL1 |
| sll1743 | rplK | 2.28 | 2.10 | 2.35 | 15.10 | Large ribosomal subunit protein uL11 |
| sll1803 | rplV | 2.24 | 1.92 | 2.36 | 14.11 | Large ribosomal subunit protein uL22 |
| ssr1398 | rpmG | 1.57 | 3.41 | 1.74 | 14.04 | Large ribosomal subunit protein bL33 |
| sll1767 | rpsF | 1.37 | 2.97 | 2.24 | 13.80 | Small ribosomal subunit protein bS6 |
| ssr1736 | rpmF | 1.09 | 3.00 | 1.70 | 10.21 | Large ribosomal subunit protein bL32 |
| sll1260 | rpsB | 1.73 | 1.32 | 2.02 | 8.42 | Small ribosomal subunit protein uS2 |
| ssl1426 | rpmI | 1.03 | 2.70 | 1.27 | 7.48 | Large ribosomal subunit protein bL35 |
| sll1821 | rplM | 1.28 | 1.34 | 1.22 | 4.90 | Large ribosomal subunit protein uL13 |
| slr1678 | rplU | 1.46 | 1.06 | 1.24 | 4.67 | Large ribosomal subunit protein bL21 |

The table presents the subset of genes whose expression level is higher or lower than log_2_1 for within the functionally enriched categories presented in table 1. The values represent the average log fold change of each gene for each starvation condition (-N, -P or -S). The genes are ranked by the value of the sum of all the pairwise multiplication products of the average fold changes (Sum of products). Gene names and descriptions sometimes change between different papers. The identifiers provided here are according to the cited references.

**Putative general stress response control and promoter sequences**

The sequences included in the table above were used as a starting point for the motif search. We analyzed the region [250 bp upstream and 50 bp downstream of the transcription start site, as described by Mitschke and co-workers (23)]. The sequences were analyzed by the MEME suite (24), restricting the limit for motifs identified was restricted to three. The distribution of these motifs within the analysed genes is presented below. The *p*-value for each gene is defined as the probability that a random sequence (with the same length and conforming to the background) would have position *p*-values such that the product is smaller or equal to the value calculated for the sequence under test.


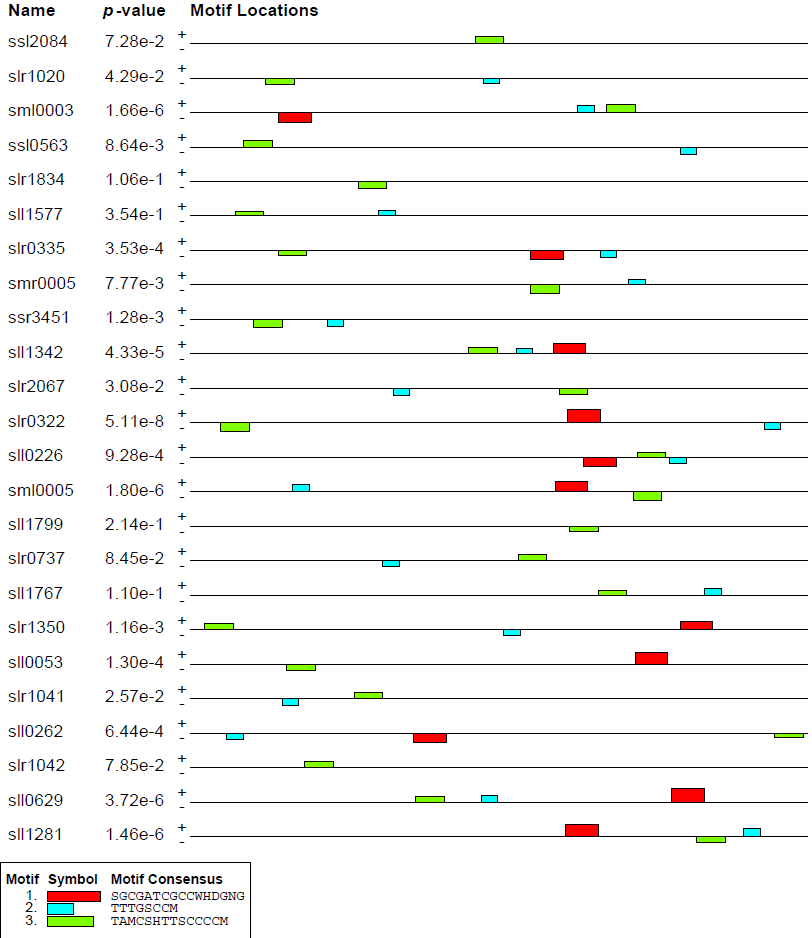

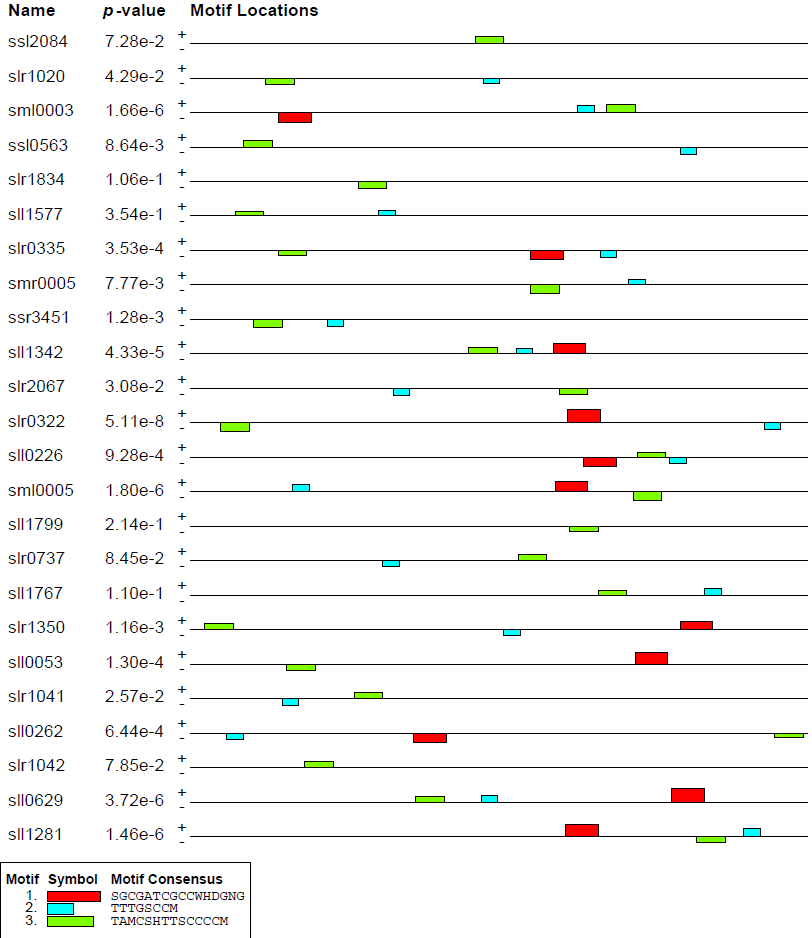


**Motif 1 - This motif appeared in 25 of the promoters analysed with an E-value of 1.2x10^-24^ which is considered as significant.**


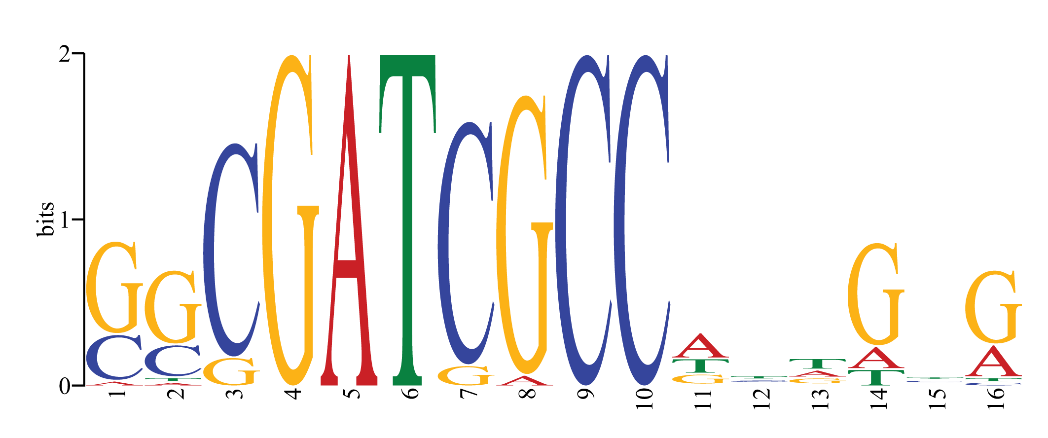


To see if this element has been previously reported it was submitted through TOMTOM. TOMTOM analysis showed that this motif resembles the binding site of 7 reported transcription factors. Being the top one AmrZ (from P. aeruginosa) which is thought to regulate the levels of c-di-GMP and through this AmrZ can act both as a positive and a negative regulator of gene expression, controlling many genes implicated in environmental adaption. Regulated traits include motility, iron homeostasis, exopolysaccharides production and the ability to form biofilms. The closest homolog in *Synechocystis* was the transcription antitermination protein NusB (sll0271) with a 34% identity.

**Motif 2 - This motif appeared in 46 of the promoters analysed with an E-value of 0.00011 which is considered significant.**


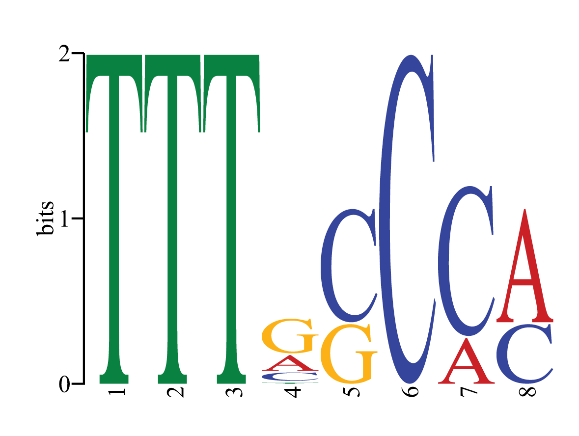


TOMTOM analysis showed that this motif resembles the binding site of 23 reported transcription factors. Being the top one MalR from Clostridiales (the MalR protein is generally a transcriptional repressor that regulates the expression of genes involved in maltose and maltodextrin utilization). The closest homolog in *Synechocystis* is the DNA-binding dual master transcriptional regulator RpaA (Slr0115) which has been reported as a regulator of PSI monomer accumulation (He et al. 2012).

**Motif 3. -This motif appeared in all promoters analysed with an E-value of 0.24 which is considered not significant.**


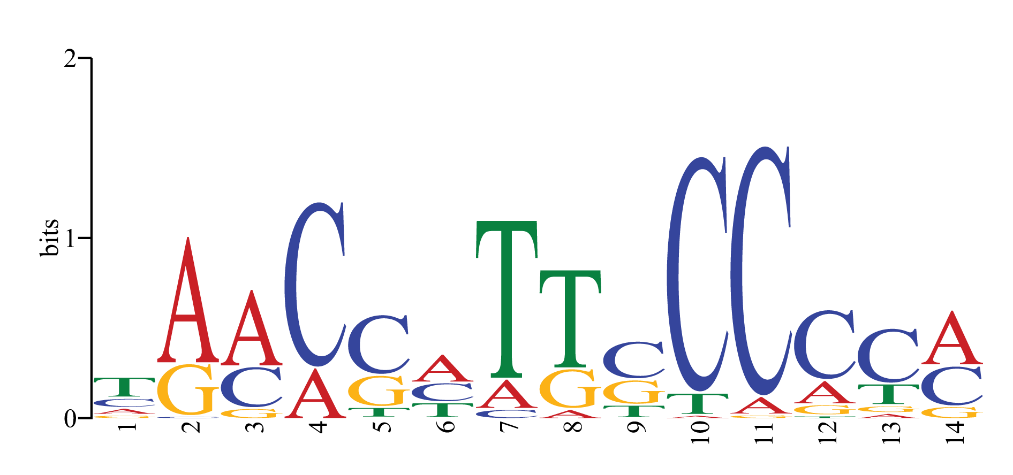


This motif matched with 18 regulatory motifs previously described in Prokaryotes. The most similar and previously reported regulatory site corresponds to genes regulated by GalR in E. coli (galactose metabolism). The next step it is to try to identify a homologous gene in Synechocystis, with the premise that if a gene is conserved also the binding site will be conserved. BLAST analysis of the GalR sequence against Synechocystis genome resulted in the identification of several low homology proteins with the top one (30% homology) being *sll0659*. The protein coded by this gene was predicted to be involved in carotenoids synthesis. However, a knockout strain did not show any significant change in carotenoid content, only some abnormality in cell structure (25).

**Identification of differentially expressed noncoding RNAs**

A number of ncRNAs that were differentially expressed, albeit not under all three conditions were identified in our study. NsiR4 (nitrogen stress-induced RNA 4 (26)), NsiR7 (nitrogen stress-induced RNA 7), ssrR (or ssaA) and PsrR1 (photosynthesis regulatory RNA1 (27)) expression levels decreased after the addition of N and S, while the expression of PmgR1 (photomixotrophic growth RNA1 (28)) increased after N or S repletion. The expression of PmgR1 have been shown to result in a reduced accumulation of glycogen indicating that under N and S depletion the cell favored glycogen accumulation. PmgR1 is also essential for photomixotrophic growth thus, its role is key when the photosynthetic machinery is impaired (28). NsiR7 has also been reported to be induced in carbon limiting conditions, playing a role, also, in the C/N balance of the cell. NsiR4 interacts with the 5’UTR of the mRNA encoding PirA interfering with its translation. PirA competes with PII which activates the key enzyme of arginine synthesis, N-acetyl-L-glutamate-kinase (29). Therefore, NsiR4 expression promotes arginine synthesis. Interestingly, previous publications have shown that NsiR4 and PirA are inversely regulated by the global nitrogen transcriptional regulator NtcA. SsaA was demonstrated to support a rapid acclimation to nitrogen availability assisting with the switch from group 2 sigma factors to SigA-dependent transcription. On the other hand, PsrR1 binds to the ribosome-binding site of multiple photosynthesis-related mRNAs, and it is required for RNase E-mediated processing of the PSI subunit psaL mRNA (27). Additionally, we observed expression over their sense mRNA of the antisense RNAs (asRNAs) for the flavodiiron protein Flv4 (sll0217), for the aluminium resistance protein Slr0850, and IsiA (sll0247) under all conditions indicating that neither Flv4, Slr0850, or IsiA play a role on the adaptation to these conditions (30). Supplemental figure 2 summarizes the suggested roles of these ncRNAs in nutrient deprivation response.

Besides these ncRNAs which have been well characterised, we identified some novel ncRNAs which were not described. Such as ncrSNP1, a newly predicted non-coding RNA, upregulated under S, N, and P limitation (Supplemental figure 3). ncrSNP1 is located at the 3’UTR of the gene ssr2153 encoding for a hypothetical protein. Using the web-based software provided through IntaRNA we identified potential pairing targets, and these were significantly enriched in genes belonging to the functional category “translation” including the sigma factor E (Supplemental figure 3D) which could point to a similar role to that observed for ssaA.


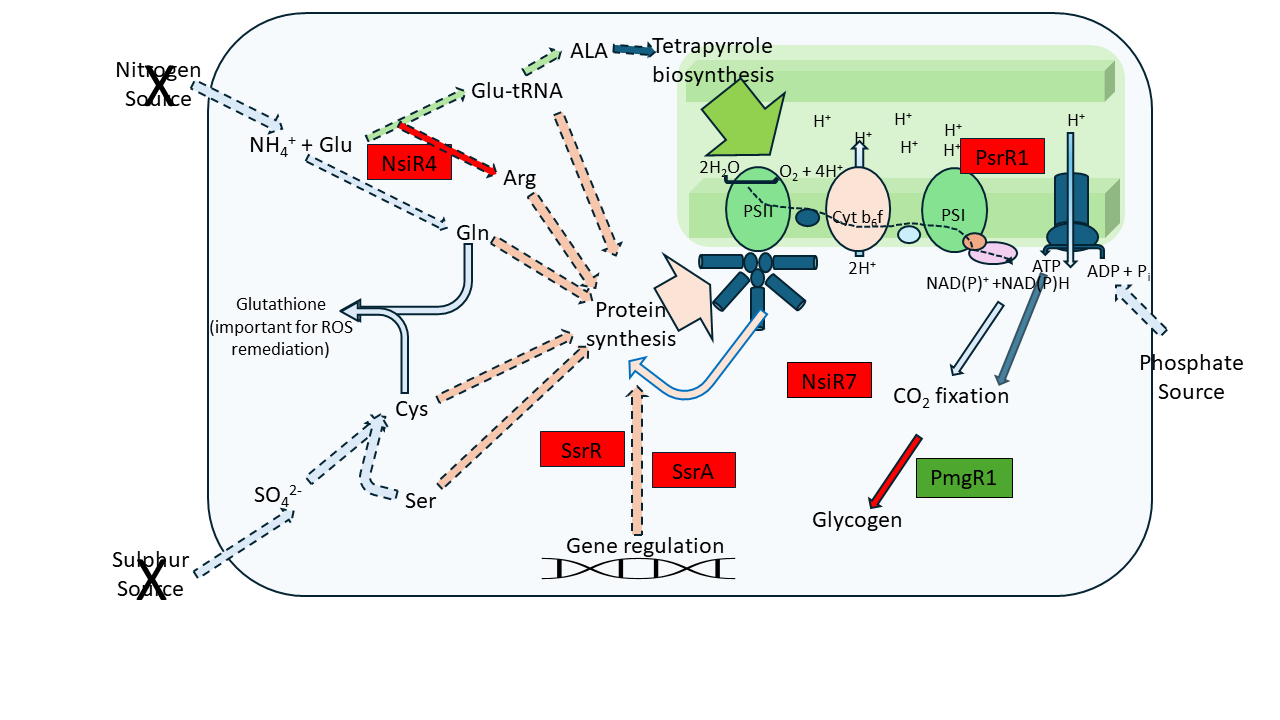


**Supplemental Figure 2: Expression of non-coding RNAs mapped onto relevant metabolic pathways during nutrient stress.**

The figure illustrates the integration of highly expressed non-coding RNAs (ncRNAs) with major metabolic and photosynthetic pathways affected under nitrogen (N) and sulfur (S) limitation. Each ncRNA is positioned according to its proposed or known functional association with specific cellular processes. Text boxes representing ncRNAs are color-coded based on their expression pattern: red indicates high expression under N and S limitation, while green indicates downregulation under these conditions. The mapping highlights the potential regulatory roles of specific ncRNAs in modulating photosynthesis, pigment biosynthesis, ribosome recycling, and general stress response pathways during nutrient deprivation. Data is based on transcriptomic analysis at the end of the limitation period (Time 0h), prior to nutrient re-addition.

**
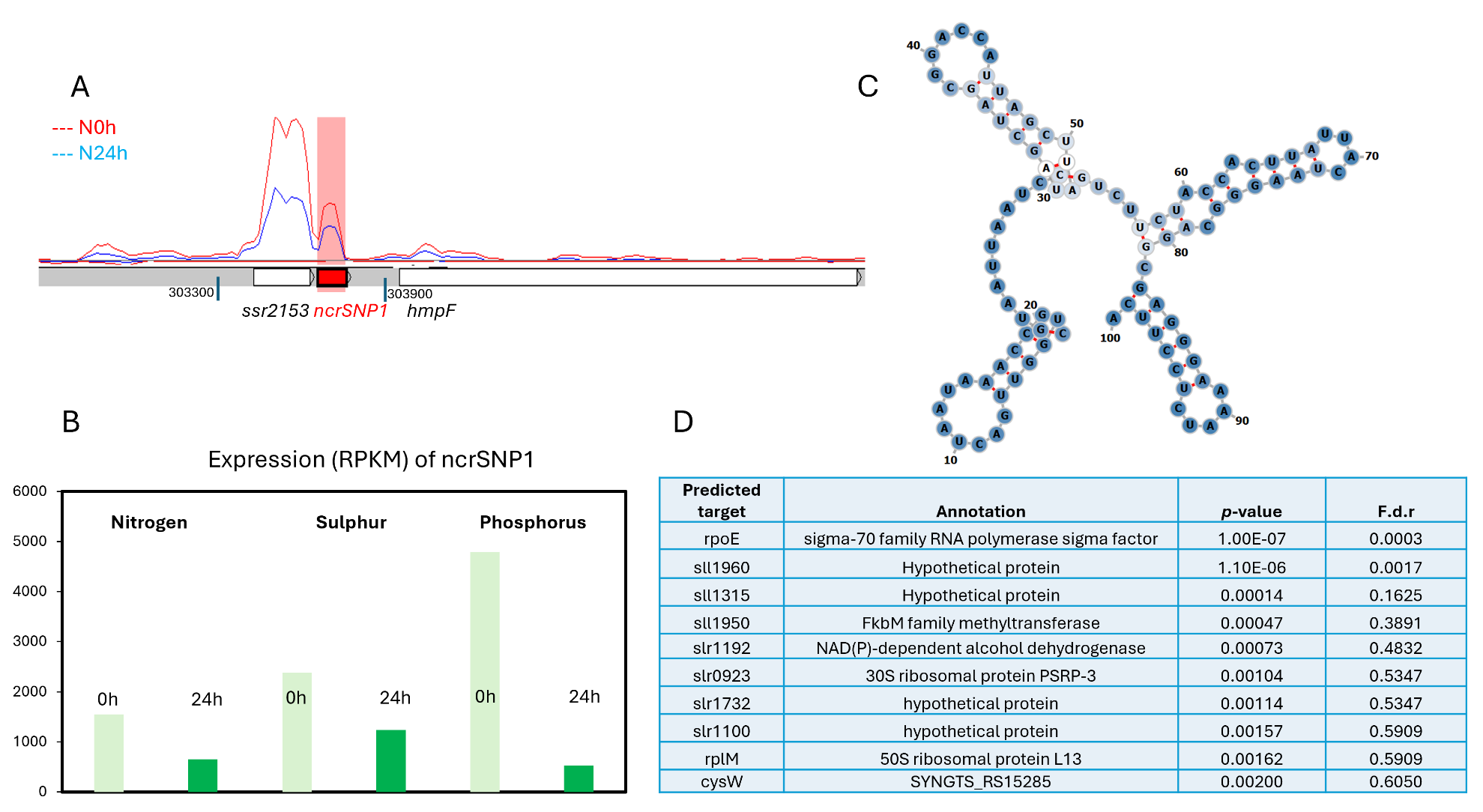
Supplemental Figure 3. Genomic context, expression dynamics, structure, and predicted targets of ncrSNP1**.

(A) Chromosomal location of ncrSNP1 in Synechocystis sp. PCC 6803, with RNA-seq read coverage shown in red for 0 h under nitrogen starvation and in blue for 24 h after nitrogen repletion. (B) Histogram showing ncrSNP1 expression levels (Kilobase of transcript per Million mapped reads - RPKM) under nitrogen (N), sulfur (S), and phosphorus (P) limitation, highlighting its upregulation under all three nutrient stresses. (C) Predicted secondary structure of ncrSNP1, generated using the RNAfold web server (D) Table of predicted mRNA targets of ncrSNP1 obtained from IntaRNA, including associated p-values and false discovery rates (FDR).

**Reference**

1. Price, G. D., Pengelly, J. J. L., Forster, B., Du, J., Whitney, S. M., von Caemmerer, S., Badger, M. R., Howitt, S. M., and Evans, J. R. (2013) The cyanobacterial CCM as a source of genes for improving photosynthetic CO2 fixation in crop species. *Journal of Experimental Botany*. **64**, 753–768

2. Impact of Carbon Fixation, Distribution and Storage on the Production of Farnesene and Limonene in Synechocystis PCC 6803 and Synechococcus PCC 7002 [online] https://www.mdpi.com/1422-0067/25/7/3827 (Accessed March 19, 2025)

3. Koksharova, O., Schubert, M., Shestakov, S., and Cerff, R. (1998) Genetic and biochemical evidence for distinct key functions of two highly divergent GAPDH genes in catabolic and anabolic carbon flow of the cyanobacterium Synechocystis sp. PCC 6803. *Plant Mol Biol*. **36**, 183–194

4. Castro-Torres, E., Jimenez-Sandoval, P., Fernández-de Gortari, E., López-Castillo, M., Baruch-Torres, N., López-Hidalgo, M., Peralta-Castro, A., Díaz-Quezada, C., Sotelo-Mundo, R. R., Benitez-Cardoza, C. G., Espinoza-Fonseca, L. M., Ochoa-Leyva, A., and Brieba, L. G. (2018) Structural Basis for the Limited Response to Oxidative and Thiol-Conjugating Agents by Triosephosphate Isomerase From the Photosynthetic Bacteria Synechocystis. *Front. Mol. Biosci.* 10.3389/fmolb.2018.00103

5. Kis, M., Zsiros, O., Farkas, T., Wada, H., Nagy, F., and Gombos, Z. (1998) Light-induced expression of fatty acid desaturase genes. *Proceedings of the National Academy of Sciences*. **95**, 4209–4214

6. Kizawa, A., Kawahara, A., Takashima, K., Takimura, Y., Nishiyama, Y., and Hihara, Y. (2017) The LexA transcription factor regulates fatty acid biosynthetic genes in the cyanobacterium Synechocystis sp. PCC 6803. *The Plant Journal*. **92**, 189–198

7. Hauf, W., Schmid, K., Gerhardt, E. C. M., Huergo, L. F., and Forchhammer, K. (2016) Interaction of the Nitrogen Regulatory Protein GlnB (PII) with Biotin Carboxyl Carrier Protein (BCCP) Controls Acetyl-CoA Levels in the Cyanobacterium Synechocystis sp. PCC 6803. *Front. Microbiol.* 10.3389/fmicb.2016.01700

8. Nelson, N. (2024) Investigating the Balance between Structural Conservation and Functional Flexibility in Photosystem I. *International Journal of Molecular Sciences*. **25**, 5073

9. Nellaepalli, S., Kim, R. G., Grossman, A. R., and Takahashi, Y. (2021) Interplay of four auxiliary factors is required for the assembly of photosystem I reaction center subcomplex. *The Plant Journal*. **106**, 1075–1086

10. Thornton, L. E., Roose, J. L., Pakrasi, H. B., and Ikeuchi, M. (2005) The Low Molecular Weight Proteins of Photosystem II. in *Photosystem II: The Light-Driven Water:Plastoquinone Oxidoreductase* (Wydrzynski, T. J., Satoh, K., and Freeman, J. A. eds), pp. 121–137, Springer Netherlands, Dordrecht, 10.1007/1-4020-4254-X_7

11. Morais, F., Barber, J., and Nixon, P. J. (1998) The Chloroplast-encoded α Subunit of Cytochrome*b-*559 Is Required for Assembly of the Photosystem Two Complex in both the Light and the Dark in *Chlamydomonas reinhardtii* *. *Journal of Biological Chemistry*. **273**, 29315–29320

12. Shen, J.-R., Burnap, R. L., and Inoue, Y. (1995) An Independent Role of Cytochrome c-550 in Cyanobacterial Photosystem II As Revealed by Double-Deletion Mutagenesis of the psbO and psbV Genes in Synechocystis sp. PCC 6803. *Biochemistry*. **34**, 12661–12668

13. Thornton, L. E., Ohkawa, H., Roose, J. L., Kashino, Y., Keren, N., and Pakrasi, H. B. (2004) Homologs of Plant PsbP and PsbQ Proteins Are Necessary for Regulation of Photosystem II Activity in the Cyanobacterium Synechocystis 6803[W]. *The Plant Cell*. **16**, 2164–2175

14. Kashino, Y., Lauber, W. M., Carroll, J. A., Wang, Q., Whitmarsh, J., Satoh, K., and Pakrasi, H. B. (2002) Proteomic Analysis of a Highly Active Photosystem II Preparation from the Cyanobacterium Synechocystis sp. PCC 6803 Reveals the Presence of Novel Polypeptides. *Biochemistry*. **41**, 8004–8012

15. Sui, S.-F. (2021) Structure of Phycobilisomes. *Annual Review of Biophysics*. **50**, 53–72

16. Watanabe, M., and Ikeuchi, M. (2013) Phycobilisome: architecture of a light-harvesting supercomplex. *Photosynth Res*. **116**, 265–276

17. Ochoa de Alda, J. A. G., and Houmard, J. (2000) Genomic survey of cAMP and cGMP signalling components in the cyanobacterium Synechocystis PCC 6803. *Microbiology*. **146**, 3183–3194

18. Okada, K., Horii, E., Nagashima, Y., Mitsui, M., Matsuura, H., Fujiwara, S., and Tsuzuki, M. (2015) Genes for a series of proteins that are involved in glucose catabolism are upregulated by the Hik8-cascade in Synechocystis sp. PCC 6803. *Planta*. **241**, 1453–1462

19. Singh, A. K., Elvitigala, T., Bhattacharyya-Pakrasi, M., Aurora, R., Ghosh, B., and Pakrasi, H. B. (2008) Integration of Carbon and Nitrogen Metabolism with Energy Production Is Crucial to Light Acclimation in the Cyanobacterium Synechocystis. *Plant Physiology*. **148**, 467–478

20. Kadowaki, T., Nishiyama, Y., Hisabori, T., and Hihara, Y. (2015) Identification of OmpR-Family Response Regulators Interacting with Thioredoxin in the Cyanobacterium Synechocystis sp. PCC 6803. *PLOS ONE*. **10**, e0119107

21. Yoshihara, S., Geng, X., and Ikeuchi, M. (2002) pilG Gene Cluster and Split pilL Genes Involved in Pilus Biogenesis, Motility and Genetic Transformation in the Cyanobacterium Synechocystis sp. PCC 6803. *Plant and Cell Physiology*. **43**, 513–521

22. Moore, K. R., Magnabosco, C., Momper, L., Gold, D. A., Bosak, T., and Fournier, G. P. (2019) An Expanded Ribosomal Phylogeny of Cyanobacteria Supports a Deep Placement of Plastids. *Front. Microbiol.* 10.3389/fmicb.2019.01612

23. An experimentally anchored map of transcriptional start sites in the model cyanobacterium Synechocystis sp. PCC6803 | PNAS [online] https://www.pnas.org/doi/abs/10.1073/pnas.1015154108 (Accessed August 21, 2025)

24. Bailey, T. L., Johnson, J., Grant, C. E., and Noble, W. S. (2015) The MEME Suite. *Nucleic Acids Res*. **43**, W39–W49

25. Liang, C.-W., Zhang, X.-W., Tian, L., and Qin, S. (2008) Functional characterization of sll0659 from Synechocystis sp. PCC 6803. *Indian J Biochem Biophys*. **45**, 275–277

26. The sRNA NsiR4 is involved in nitrogen assimilation control in cyanobacteria by targeting glutamine synthetase inactivating factor IF7 | PNAS [online] https://www.pnas.org/doi/10.1073/pnas.1508412112 (Accessed June 16, 2025)

27. Georg, J., Dienst, D., Schürgers, N., Wallner, T., Kopp, D., Stazic, D., Kuchmina, E., Klähn, S., Lokstein, H., Hess, W. R., and Wilde, A. (2014) The Small Regulatory RNA SyR1/PsrR1 Controls Photosynthetic Functions in Cyanobacteria. *The Plant Cell*. **26**, 3661–3679

28. de Porcellinis, A. J., Klähn, S., Rosgaard, L., Kirsch, R., Gutekunst, K., Georg, J., Hess, W. R., and Sakuragi, Y. (2016) The Non-Coding RNA Ncr0700/PmgR1 is Required for Photomixotrophic Growth and the Regulation of Glycogen Accumulation in the Cyanobacterium Synechocystis sp. PCC 6803. *Plant and Cell Physiology*. **57**, 2091–2103

29. Bolay, P., Rozbeh, R., Muro-Pastor, M. I., Timm, S., Hagemann, M., Florencio, F. J., Forchhammer, K., and Klähn, S. (2021) The Novel PII-Interacting Protein PirA Controls Flux into the Cyanobacterial Ornithine-Ammonia Cycle. *mBio*. **12**, 10.1128/mbio.00229-21

30. Georg, J., Voß, B., Scholz, I., Mitschke, J., Wilde, A., and Hess, W. R. (2009) Evidence for a major role of antisense RNAs in cyanobacterial gene regulation. *Molecular Systems Biology*. **5**, 305
